# Pedigree reconstruction from poor quality genotype data

**DOI:** 10.1038/s41437-018-0178-7

**Published:** 2019-01-10

**Authors:** Jinliang Wang

**Affiliations:** Institute of Zoology, Zoological Society of London, London, NW1 4RY UK

**Keywords:** Population genetics, Molecular ecology

## Abstract

Marker genotype data could suffer from a high rate of errors such as false alleles and allelic dropouts (null alleles) in situations such as SNPs from low-coverage next-generation sequencing and microsatellites from noninvasive samples. Use of such data without accounting for mistyping properly could lead to inaccurate or incorrect inferences of family relationships such as parentage and sibship. This study shows that markers with a high error rate are still informative. Simply discarding them could cause a substantial loss of precious information, and is impractical in situations where virtually all markers (e.g. SNPs from low-coverage next-generation sequencing, microsatellites from noninvasive samples) suffer from a similarly high error rate. This study also shows that some previous error models are valid for markers of low error rates, but fail for markers of high error rates. It proposes an improved error model and demonstrates, using simulated and empirical data of a high error rate (say, >0.5), that it leads to more accurate sibship and parentage inferences than previous models. It suggests that, in reality, markers of high error rates should be used rather than discarded in pedigree reconstruction, so long as the error rates can be estimated and used properly in the analyses.

## Introduction

Molecular marker data are widely used to reconstruct pedigrees of wild populations in which behaviour data are difficult to obtain (Pemberton 2008). The complete, or most often partial, pedigrees recovered from marker data could then be used to make evolutionary and conservation studies of populations in their natural habitats. Such studies include investigating, for example, the mating system and reproductive success, inbreeding and inbreeding depression, the inheritance of qualitative (e.g. some genetic diseases or abnormalities) and quantitative (e.g. heritability of body weight) traits, and the conservation management of endangered species (e.g. for minimizing loss of genetic diversity). Indeed, molecular pedigrees have contributed tremendously to our understanding and the management of wild species. For example, many socially monogamous bird species are found to have extra-pair paternity (EPP) from genetic marker-based parentage analyses, and the EPP rates range up to 55% across species and between populations within species (Griffith et al. 2002).

Like other types of data, however, molecular marker data are not perfect. In various situations, marker data quality can be rather poor, due to many factors such as genotyping errors (e.g. allelic dropouts), mutations, imperfect markers or genotyping technology (e.g. null or recessive alleles which have no phenotypes) and missing data (Bonin et al. 2004; Pompanon et al. 2005). It has long been recognized that such quality problems have large impacts on genealogical relationship inferences (e.g. Sobel et al. 2002; Douglas et al. 2002), and, if ignored, could cause grossly erroneous inferences. Parentage assignments are particularly vulnerable to genotyping problems, as a parent−offspring pair must share at least one allele identical in state at each locus. A single genotyping error in the pair could cause a genotype mismatch and thus a false parentage exclusion, no matter how many other loci are correctly genotyped and are thus in support of the relationship. Recognizing these, various genotyping error models (e.g. Ott 1993; Ehm et al. 1996; Sobel et al. 2002; Marshall et al. 1998; Wang 2004; Hadfield et al. 2006) have been proposed and integrated into likelihood methods for robust relationship inference. Wang (2004) showed that, with an increasing number of markers suffering from genotyping errors at a low rate, sibship inference quality declines rapidly if the errors are ignored but improves rapidly if they are integrated into the likelihood inference framework, respectively.

Previous work focussed on modelling and evaluating the impacts of genotyping errors occurring at a relatively small rate (say, <5%). Such “high” quality data can be obtained from microsatellites genotyped by optimized PCR (polymerase chain reaction) using DNA templates of sufficiently high quality and quantity (e.g. extracted from fresh tissue or blood samples), or from SNPs genotyped by microarrays or from high-coverage next-generation sequencing (NGS) technologies. However, much dirtier data with a high genotyping error rate are now widely generated and used in molecular ecology, evolution, and conservation studies. First, SNPs from low-coverage NGS are now regularly generated for many species. Such data can have an extremely high frequency of false homozygotes produced by random sampling of the two alleles in a heterozygote (Nielsen et al. 2011). A heterozygote (say, *cg*) would be determined to be a homozygote (*cc* or *gg*) with probabilities 75, 54, 36 and 24% when NGS has an average sequencing coverage of 1, 2, 3, and 4 respectively (see below). Second, microsatellites genotyped by PCR from noninvasive samples (e.g. faeces, hair) can also show a high frequency of erroneous genotypes because of multiple causes such as low quantity and quality of DNA, pollution, and the presence of PCR inhibitors (Pompanon et al. 2005). Genotyping errors occurring at a high rate of 37% per locus has been reported (Gagneux et al. 1997) for microsatellites genotyped from single shed hair in wild chimpanzees.

There are several outstanding questions to be asked and answered about pedigree reconstruction from marker data with high genotyping error rates. First, are current error models valid in handling data with high error rates? Second, what is the highest error rate that current pedigree reconstruction methods can tolerate and thus a marker with such a high error rate is still informative and useful? Cautious researchers usually discard markers with a moderate error rate (say >7%, Hauser et al. 2011) in pedigree analyses. However, these threshold error rates are more or less arbitrary and could be either too low, wasting valuable marker information, or too high, potentially derailing a pedigree analysis.

This study aims to address these questions. I will show that current error models in marker-based pedigree reconstruction can adequately handle markers with a small to moderate genotyping error rate. However, these models fail for markers with a high error rate and lead to erroneous inferences. I will develop an improved error model that is valid and powerful for markers with very high error rates (say, >50%). The old and new models are then checked and compared in inferring full sibship, half sibship and parentage from simulated SNPs of a high dropout rate (mimicking low-coverage NGS) or a high false allele rate, and from simulated microsatellites of a high false allele rate (mimicking PCR of low-quality noninvasive samples). Their performances are also demonstrated by analysing a real dataset. In conclusion, the study advocates the wide application of the new error model, and encourages using rather than discarding markers of poor genotyping quality in marker-based pedigree analysis and other analyses of individual genotypes.

## Methods

Throughout the paper, I will use uppercase letters to denote genotypes or true genealogical relationships, and use lowercase letters to denote phenotypes or inferred genealogical relationships.

### Error models

Through different mechanisms, mutations, imperfection of markers or genotyping technology (e.g. recessive or null alleles), and genotyping errors could all cause an observed genotype (phenotype) to differ from its underlying true genotype. They have the same effect on data quality and cause essentially the same consequence in inferences. Therefore these problems are not distinguished and are collectively called typing errors hereafter. The essence of an error model is to consider the uncertainty of a phenotype by calculating and using the probabilities of its different underlying genotypes for a given mistyping rate. Following previous studies on pedigree reconstruction (e.g. Wang 2004), I consider two error models detailed below.

### Allelic dropouts

Wang (2004) proposed an error model to handle false homozygotes generated by allelic dropouts of microsatellites during PCR. An allelic dropout occurs when PCR fails to amplify one of an individual’s two homologous genes at a locus, leading to a false homozygote phenotype when the underlying genotype is a heterozygote. The model can also handle false homozygotes due to other causes, such as those produced by null alleles (Wang 2018) or by sampling errors in SNP data from low-coverage NGS.

The error model assumes that each of the two homologous genes in a diploid individual drops out during PCR at the same rate *ε*_1_, and that double dropouts of both genes do not occur (Wang 2004). For a heterozygote genotype *G* = *A*_1_*A*_2_, the model leads to Prb(*g*|*G*) = 1− 2*e*_1_, *e*_1_ and *e*_1_ when the observed phenotype is *g* = *A*_1_*A*_2_, *g* = *A*_1_*A*_1_ and *g* = *A*_2_*A*_2_, respectively, where *e*_1_ = *ε*_1_/(1 + *ε*_1_). For a homozygote genotype *G* = *A*_*i*_*A*_*i*_, the model leads to Prb(*g*|*G*) = 1 and 0 when *G* is observed to be *g* = *A*_*i*_*A*_*i*_ and any other phenotypes, respectively. Null alleles can also be handled by this error model and the parameter *ε*_1_ is equivalent to null allele frequency (Wang 2018).

### False alleles

In contrast to allelic dropouts that affect heterozygotes only and cause an apparent homozygote excess, the false allele model applies to any genotypes, homozygotes or heterozygotes. It makes two assumptions. First, the two homologous genes at a locus in a diploid individual are independently and equally likely to be incorrectly observed, with rate *ε*_2_. Second, when an allele is incorrectly observed, it is observed to be any one of the other alleles at an equal probability of *e*_2_ = *ε*_2_/(*k* − 1), where *k* is the observed number of alleles at a focal locus (Wang 2004, 2018). Thus the probabilities that an allele X is observed as such (x) and as one of the other alleles (y, y ≠ x) at the locus are Prb(x|X) = 1 − *ε*_2_ and Prb(y|X) = *e*_2_ = *ε*_2_/(*k* − 1), respectively. These probabilities sum to 1, Prb(x|X) + (*k* − 1)Prb(y|X) ≡ 1, as expected.

The above model proves to be powerful in accounting for mistypings in marker data for sibship and parentage inferences (Wang 2004; Wang and Santure 2009; Hadfield et al. 2006), and in inferring the mistyping rate *ε*_2_ at a locus given a reconstructed pedigree (Wang 2018). However, this is true only when *ε*_2_ is not very high (say, <0.3) and *k* is not small. Otherwise, the model could lead to, counterintuitively, a decreasing uncertainty of a phenotype with an increasing value of *ε*_2_, and thus suboptimal or incorrect results. To understand this, consider the case of *k* = 2. According to the above error model, the probabilities that an allele X is observed to be itself, x, and the alternative allele, y, are Prb(x|X) = 1 − *ε*_2_ and Prb(y|X) = *ε*_2_, respectively. With an increasing value of *ε*_2_ > 0.5, Prb(x|X) becomes increasingly smaller than Prb(y|X). In the extreme case of *ε*_2_ = 1, Prb(x|X) = 0 and Prb(y|X) = 1. This problem as exemplified above yields two counterintuitive results. First, an observation becomes increasingly less uncertain when *ε*_2_ gets closer to 1. Second, an observed allele is inferred to be more likely to be any one of the other alleles than the observed one, and this probability increases to 1 when *ε*_2_ increases to 1. Although this problem diminishes with an increasing *k*, it does not disappear completely even when *k* is large. In the literature, quite a few error models with subtle differences were proposed (e.g. Sancristobal and Chevalet 1997; Sieberts et al. 2001; Sobel et al. 2002), but all suffer from this type of problems.

An improved model for false alleles is to change the second assumption to that, when an allele mutates or is mistyped, it is observed to be any allele, including itself, at an equal probability of *e*_2_ = *ε*_2_/*k*. In other words, an observed allele is independent of the underlying true allele when a mistyping occurs. Thus the probabilities that an allele X is observed as itself, x, and as one of the other alleles, y (y ≠ x), at the locus are Prb(x|X) = 1 − *ε*_2_ + *e*_2_ and Prb(y|X) = *e*_2_, respectively, where *e*_2_ = *ε*_2_/*k*. These probabilities sum to 1, Prb(x|X) + (*k* − 1)Prb(y|X) ≡ 1, as expected. This error model ensures that the probability an allele is correctly observed is always not smaller than that it is incorrectly observed to an alternative allele. With *ε*_2_ increasing towards the limiting value of 1, the uncertainty of an observation always increases, and Prb(x|X) always decreases but is never smaller than Prb(y|X). For the case of *k* = 2, Prb(x|X) = 1 − *ε*_2_/2 and Prb(y|X) = *ε*_2_/2. The minimum value (0.5) of Prb(x|X) is equal to the maximum value (0.5) of Prb(y|X), which occurs at the highest possible value of *ε*_2_ = 1. This error model was used in evaluating marker information content (Wang 2006), and some variants were also used in detecting mistypings in pedigree genotyping data (e.g. Ehm et al. 1996).

### Error-penetrance function

Given the two error (dropouts and false alleles) models and error rates (*ε*_1_, *ε*_2_), it is straightforward to derive the probability that a genotype is observed to be any phenotype. These error-penetrance functions were shown in Wang (2004) for the above dropout model and the old false allele model. They are good approximations when the rates of both types of errors are small, but become increasingly inappropriate and could cause a loss of relationship inference accuracy when error rates are substantial (as shown below).

For the improved false allele model, these error-penetrance functions are listed in Table 1, derived by assuming that, when both false alleles and dropouts occur to the same genotype, they occur in that order. These functions are exact regardless of the values of *ε*_1_ and *ε*_2_, and regardless of the number of alleles, *k*, at a locus. For a locus with *k* codominant alleles, there are *k*(*k* + 1)/2 possible genotypes and the same number of phenotypes. The probabilities of these phenotypes for any given genotype always sum to 1, which can be confirmed by using the functions listed in Table 1.

**Table 1:** Error-penetrance functions

Genotype	Phenotype	Penetrance function (Prb(*g*|*G*))
*G*_*st*_	*g*_*st*_	$\left( {\left( {1 - \varepsilon _2 + e_2} \right)^2 + \, e_2^2} \right)\left( {1 - \varepsilon _1} \right)/\left( {1 + \varepsilon _1} \right)$
*G*_*st*_	*g*_*ss*_ or *g*_*tt*_	((*ε*_1_ + (1 − *ε*_1_)*e*_2_)*e*_2_(1 − *k*) + *ε*_1_ + *e*_2_)/(1 − *ε*_1_)
*G*_*st*_	*g*_*uu*_	*e*_2_(*e*_2_ − *ε*_1_*e*_2_ + 2*ε*_1_)/(1 + *ε*_1_)
*G*_*st*_	*g*_*uv*_	$2e_2^2\left( {1 - \varepsilon _1} \right)/\left( {1 + \varepsilon _1} \right)$
*G*_*st*_	*g*_*su*_ or *g*_*tu*_	(1 − *ε*_2_ + 2*e*_2_)*e*_2_(1 − *ε*_1_)/(1 + *ε*_1_)
*G*_*ss*_	*g*_*ss*_	(1 − *ε*_2_ + *e*_2_)^2^ + 2(1 − *ε*_2_ + *e*_2_)(*ε*_2_ − *e*_2_)*e*_1_
*G*_*ss*_	*g*_*st*_	2(1 − *ε*_2_ + *e*_2_)*e*_2_(1 − *ε*_1_)/(1 + *ε*_1_)
*G*_*ss*_	*g*_*tt*_	$2e_2\left( {1 - e_2} \right)e_1 + e_2^2$
*G*_*ss*_	*g*_*tu*_	$2e_2^2\left( {1 - \varepsilon _1} \right)/\left( {1 + \varepsilon _1} \right)$

The third column gives the probability of a phenotype (second column, *g*) given a genotype (first column, *G*), Prb(*g|G*). A locus has *k* codominant alleles. Allele indexes *s, t, u, v* indicate different alleles. Allelic dropout rate and false allele rate are *ε*_1_ and *ε*_2_ respectively, and *e*_1_ = *ε*_1_/(1 + *ε*_1_), *e*_2_ = *ε*_2_/*k*

### Simulated pedigrees

There are so many possible two-generation pedigrees in reality that even a simulation study can only consider a few, hopefully representative, ones. In general, half sibship (HS) is much more difficult to infer than full sibship (FS) and parent−offspring relationship (PO), because genotypes of HS have more uncertainties in IBD (identical by descent) sharing and HS is difficult to distinguish from unrelated (UR). The focus of this study is to investigate whether the current and the improved error models can handle high mistyping rates, and whether markers of extremely high error rates are still informative for relationship inference and thus should be used rather than discarded or not. Therefore, I choose to analyse two pedigrees with modest difficulty to infer.

The first pedigree contains eight sets of full sibships, with set *i* (*i* = 1~8) having 2^8−*i*^ identical sibships and each sibship having 2^*i*−1^ offspring coming from the same pair of parents. The sampled pedigree has thus a total number of 1024 offspring distributed in 255 full sibships, many being very small (e.g. 128 sibships with each having just 1 offspring and 64 sibships with each having only 2 offspring) and a few being very large (e.g. the largest sibship has 128 offspring). Many singletons and small sibships mean a high potential of type I errors (i.e. non-sibs being inferred to be sibs), and very large sibships mean also a high chance of type II errors (i.e. sib family being split and sibs being inferred to be non-sibs).

The simulated pedigree has in total 255 sibships, each being produced by two singly mated and unrelated parents. Among the 255 true fathers, 15 are selected at random to include in a candidate father sample, which also contains 85 unrelated males. Thus the probability that a true father is sampled is about 0.06, and this probability is used in parentage analysis. The candidate mother sample is generated similarly.

The second pedigree contains seven sets of half sibships. Each set *i* (*i* = 1~7) has 2^7−*i*^ identical half sibships, with each half sibship having 2^*i*−1^ mothers mated with a single father and with each mating producing a single offspring. Similar to pedigree one, pedigree two has many small half sibships and a few large half sibships. Among the 127 true fathers, 15 are selected at random to include in a candidate father sample, which also contains 85 unrelated males. Thus the probability that a true father is sampled is about 0.12, and this probability is used in parentage analysis. Among the 448 true mothers, 15 are selected at random to include in a candidate mother sample, which also contains 85 unrelated females. Thus the probability that a true mother is sampled is about 0.03, and this probability is used in parentage analysis.

### Simulation of SNPs from NGS

I simulated a number of *L* variable loci equally spaced in a genomic region of *d* Morgans in genetic map length. Each locus was assumed to have two alleles, with allele frequencies drawn at random from a uniform distribution in the range (0, 1). Given the simulated pedigree, the genotype of each individual at each locus was simulated following Mendelian inheritance laws. Linkage and recombination was simulated by assuming no crossover interference, using Haldane’s mapping function $r = \frac{1}{2}\left( {1 - e^{ - 2d}} \right)$, where *r* is the recombination rate. For each simulated individual at each locus, the number of reads, *n*, was drawn from a Poisson distribution with the mean equal to the specified sequencing coverage, *c*. To simulate sequencing errors, the allele of each read was changed to any of the two alleles (i.e. including itself) at an equal rate *ε*_2_/2, where *ε*_2_ is the false allele rate. When *n* = 0, the genotype was determined as missing. When the *n* (>0) reads at a locus of an individual display one or two allele types, the genotype is determined as a homozygote or heterozygote of the observed alleles, respectively.

In the simulations, I assumed *L* = 100, 1000 and 10,000, *d* = 1, 5, and 10*M*, *ε*_2_ = 0.5, 1, and 2%, and *c* = 1, 2, 3, 4, 5 and 10. In analysing the data by Colony (Wang 2004; Jones and Wang 2010), the simulated value of *ε*_2_ was used as the false allele rate at a locus, and the dropout rate at a locus is calculated as

1 $$\varepsilon _1 = \frac{{\mathop {\sum }\nolimits_{n = 1}^\infty 0.5^{n - 1}c^n{\mathrm{exp}}\left( { - c} \right)/n!}}{{1 - {\mathrm{exp}}\left( { - c} \right)}} = \frac{2}{{1 + {\mathrm{exp}}\left( {c/2} \right)}}.$$

When *c* = 1, 2, 3, 4, 5 and 10, Eq. (1) gives a dropout rate of *ε*_1_ = 0.75, 0.54, 0.36, 0.24, 0.15 and 0.01 approximately.

### Simulation of SSRs from noninvasive sampling

I simulated a number of *L* unlinked microsatellites, each having *k* alleles in a uniform frequency distribution. Due to factors such as the presence of inhibitors, pollutions, and the low quality and quantity of DNA extracted from noninvasive samples (such as faeces), a high dropout rate (*ε*_1_) and a high other error rate (*ε*_2_) are possible (Gagneux et al. 1997; Pompanon et al. 2005). Because dropouts were considered in NGS data, I focused on false alleles by keeping a constant and low dropout rate of *ε*_2_ = 0.01 and by considering a wide range of *ε*_2_ values (0.01~0.64) for each locus. The values of *k* used in simulations were 10 and 2, for microsatellites and SNPs, respectively.

### Simulation of SNPs with high and variable false allele rates

I simulated data of a number (*L* = 100, 500) of unlinked SNPs with high false allele rates, *ε*_2_, to compare the accuracies of the old and new false allele models for full-sib, half-sib and parentage inferences. I also simulated unlinked SNPs with variable *ε*_2_ across loci to investigate the effects of the variation in *ε*_2_ on relationship inference. In both cases, the allelic dropout rate at each locus was fixed at 0.01. For variable *ε*_2_, I assumed that *ε*_2_ followed Beta(*α*, *β*) distribution, and a value drawn from the distribution was taken as *ε*_2_ for a locus. The mean and variance of a variable in Beta(*α*, *β*) distribution are *α*/(*α* + *β*) and *αβ*/(*α* + *β*)^2^/(*α* + *β* + 1) respectively. For a given mean $\overline {\varepsilon _2}$ and variance *v*_2_ of *ε*_2_ in Beta(*α*, *β*), therefore, the parameter values *α* and *β* can be derived as

${\mathrm{\alpha }} = \overline {\varepsilon _2} ^2\left( {1 - \overline {\varepsilon _2} } \right)/v_2 - \overline {\varepsilon _2}$ and ${\mathrm{\beta }} = \left( {\overline {\varepsilon _2} - 1} \right)\left( {\overline {\varepsilon _2} ^2 - \overline {\varepsilon _2} + v_2} \right)/v_2$, respectively. I used $\overline {\varepsilon _2}$ = 0.2 and *v*_2_ = {0.001, 0.002, 0.004, 0.008, 0.016, 0.032, 0.064} in the simulations.

### An ant dataset

This dataset was used in studying the mating frequency of an ant species, *Leptothorax acervorum* (Hammond et al. 2001). A total number of 377 ant workers (diploid) were sampled from 10 colonies, with each of 6 colonies contributing 45 workers and the remaining 4 colonies contributing 47, 44, 9, and 7 workers to the sample. Each sampled colony was known to be headed by a single (diploid) queen mated with a single (haploid) male; thus the sampled workers from the same colony and different colonies were full siblings and non-siblings respectively. Each sampled worker was genotyped at up to six microsatellite loci, which had a number of observed alleles varying between 3 and 22.

To investigate the effects of genotyping errors and the ability of different error models in handling the errors, the worker genotype data were modified by introducing each type (allelic dropouts or false alleles) of errors separately and at varying rates. For each type and rate (=0.05, 0.1, 0.15, 0.2, 0.3, 0.4, 0.5, 0.6, 0.7) of errors, a number of 100 replicate datasets were generated from the original dataset. To simulate dropouts at rate *e*, the genotype at each locus of each individual was examined. If it had a homozygote or missing genotype, then no changes were made. If it had a heterozygous genotype, then a uniformly distributed random number *R* was generated. If *R* ≤ *e*, a dropout occurred and the genotype was reset to a homozygote of one allele taken at random from the two alleles of the heterozygote. Otherwise, the genotype was not changed. Similarly, the false allele model was applied to each allele in a genotype at each locus of each worker at a rate *e* (details below). Accuracy of reconstructed pedigree was assessed by comparing the known and estimated pedigrees.

### Data simulation and analyses

For each parameter combination, a number of 100 replicate datasets were simulated and analysed by the pedigree reconstruction program Colony (Jones and Wang 2010) using either the old or/and the new error models. Note false allele rate is defined and modelled differently in the old and new models. In simulations, false alleles were generated following the new false allele model, and the simulated false allele rate, *ε*_2_, was used in analysing the data by the new error model. The same data were analysed also by the old error model, with false allele rate *ε*_2_ being reduced to $\varepsilon _2^ \ast = \left( {1 - 1/k} \right)\varepsilon _2$ for a locus with *k* alleles. In presentations of the analysis results, *ε*_2_ (rather than $\varepsilon _2^ \ast$) was used, bearing in mind that the actual value used by the old error model was $\varepsilon _2^ \ast = \left( {1 - 1/k} \right)\varepsilon _2$.

### Assessments of inference quality

The quality of reconstructed pedigrees was assessed by evaluating the frequency that a pairwise relationship was correctly inferred. Among the 1024 offspring in a sample from simulated pedigree one, there are two types of pairwise relationships, full siblings (FS) and non-siblings (NS). The accuracy measures are thus Prb(fs|FS) and Prb(ns|NS), the frequencies that a random FS and non-sib (NS) pair are correctly inferred as such (i.e. fs and ns). Between the offspring and candidate parents, there are two types of pairwise relationships, PO and unrelated (UN). I use the frequencies that a dyadic relationship (PO or UN, in a total of 1024 × 200 = 204,800 pairs) is correctly inferred, Prb(po|PO) and Prb(un|UN), to assess parentage assignment accuracy. For simulated pedigree two, there are two types of relationships among offspring, half siblings (HS) and non-siblings (NS), and two types of relationships between offspring and candidate parents, PO and unrelated (UN). Accuracy is thus measured by Prb(hs|HS), Prb(ns|NS), Prb(po|PO), and Prb(un|UN).

To measure the overall accuracy of the inferred relationship among offspring, I calculate Prb(OO) = Prb(fs|FS) × Fre(FS) + Prb(ns|NS) × Fre(NS), where Fre(FS) and Fre(NS) are the frequencies of FS pairs and NS pairs, respectively, in the offspring sample. Therefore, Prb(OO) is the frequency that the relationship of a pair of individuals drawn at random from the offspring sample is correctly inferred. For pedigree two, Prb(OO) is calculated similarly, replacing Prb(fs|FS) and Fre(FS) by Prb(hs|HS) and Fre(HS), respectively. Similarly, the overall accuracy of the inferred relationship between offspring and candidate parent samples is calculated by Prb(PO) = Prb(po|PO) × Fre(PO) + Prb(un|UN) × Fre(UN).

Accuracy is assessed similarly for the real dataset, using Prb(fs|FS) and Prb(ns|NS), as the sibship among sampled offspring is known.

## Results

### Comparison of false allele models

**Fig. 1:**
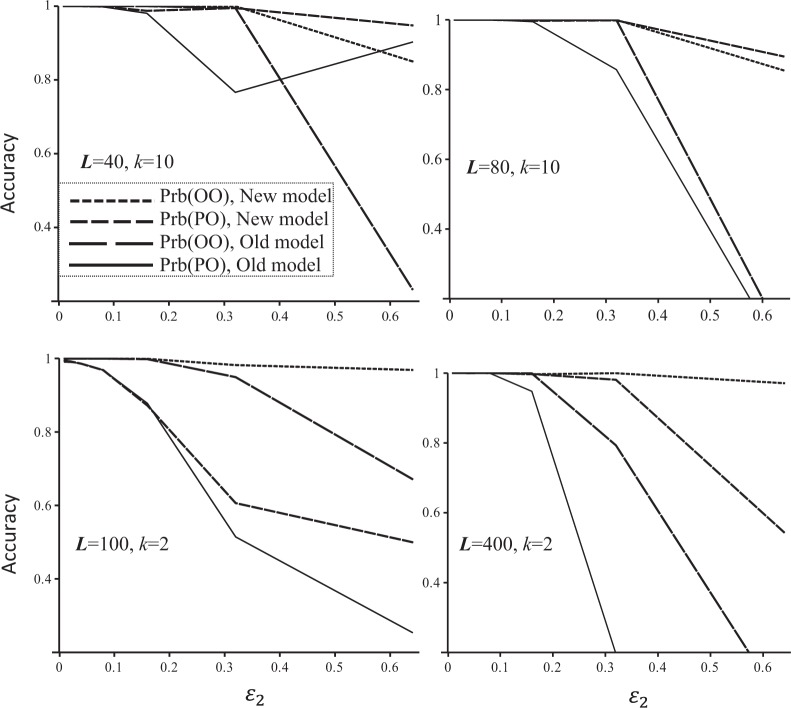
A comparison of the new and old error models for full sibship and parentage inference accuracy (Prb(OO) and Prb(PO), *y-*axis) as a function of false allele rate (*ε*_2_, *x-*axis). A number of 40 (upper left panel) or 80 (upper right panel) unlinked microsatellites, or a number of 100 (lower left panel) or 400 (lower right panel) unlinked SNPs, were used in reconstructing the simulated full-sib pedigree. Each microsatellite and SNP was assumed to have 10 and 2 alleles, respectively, in a uniform frequency distribution. The false allele rate for each marker varies from 0.01 to 0.64, while allelic dropout rate is fixed at 0.01 for each locus in each simulation

When false allele rate (*ε*_2_) is low (i.e. *ε*_2_ < 0.1), the new and old error models yield almost identical results, as is expected. This is true for both sibship and parentage assignments, no matter the markers are microsatellites (*k* = 10) or SNPs (*k* = 2) and whether the number of markers used in the inference is high (*L* = 40 and 80 for microsatellites, *L* = 100 and 400 for SNPs, Fig. 1) or low (e.g. *L* = 10 for microsatellites or *L* = 50 for SNPs, data not shown). With an increasing *ε*_2_ above 0.2, however, the new error model gives increasingly more accurate inferences than the old model (Fig. 1). The contrast between models is especially startling when both *ε*_2_ and *L* are large.

Figure 1 also demonstrates that markers of extremely high error rates (*ε*_2_) are still informative about pedigrees when the proper new error model is applied. At the high false allele rate of *ε*_2_ = 0.64 for example, the accuracy of sibship and parentage can still reach 0.9 when *L* = 40 microsatellites are used by the new error model. However, this is not true under the old error model. This means that markers of extremely high error rates are still informative for relatedness under the new error model, but are uninformative under the old error model.

**Fig. 2:**
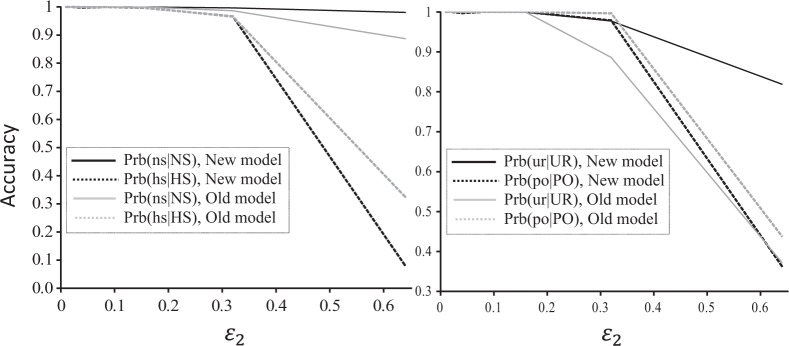
A comparison of the new and old error models for half sibship and parentage inference accuracy as a function of false allele rate. A number of 500 SNPs, each with a fixed dropout rate of 0.01 and a variable false allele rate (*ε*_2_, *x-* axis), were used for reconstructing the simulated half-sib pedigree. Each locus was assumed to have alleles in a uniform frequency distribution

Similar results were obtained in using SNPs of various false allele rates for reconstructing half-sib families (Fig. 2). Again the new and old error models are indistinguishable when *ε*_2_ < 0.2. With increasing values of *ε*_2_ > 0.2, the new model becomes more accurate in inferring non-sibs (NS) among offspring and in identifying unrelated (UR) pairs of offspring and candidate parents, while the old model becomes more accurate in inferring half-sibs (HS) among offspring and in inferring parentage (PO). However, because NS and UR relationships are almost always much more frequent than HS and PO relationships in a dataset, the overall accuracy measured by the frequency that the relationship of a random pair of individuals is correctly inferred is higher for the new model than the old model. Basically, the old model results in over-assignments of HS and PO relationships, and infers many false HS and PO relationships.

### SNP data of different dropout rates

**Fig. 3:**
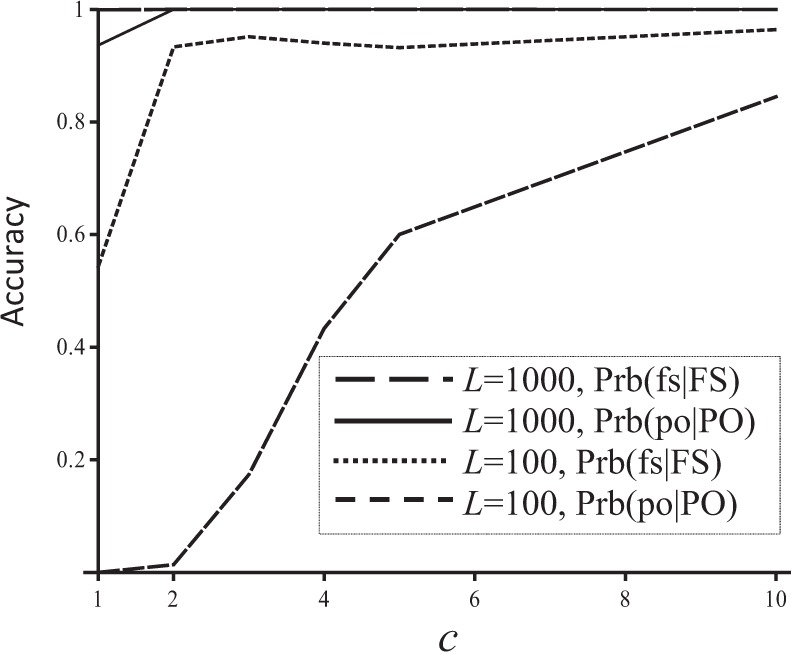
Sibship and parentage inference accuracy (Prb(fs|FS) and Prb(po|PO), *y-*axis) as a function of average coverage (*c*, *x-*axis). A number of 100 or 1000 SNPs distributed evenly on a chromosome segment of 5 Morgans in genetic map length were used in reconstructing the simulated full-sib pedigree, with each SNP having two alleles of frequencies drawn from a uniform distribution. The sequencing error rate for each read was 0.01. Prb(ns|NS) and Prb(un|UN) are always close to 1 and are thus not plotted for clarity

The accuracy of both sibship and parentage assignments increases with an increasing average coverage, *c* (Fig. 3). While Prb(fs|FS) quickly reaches a plateau (at 0.94) at a low coverage of *c* = 2, Prb(po|PO) increases with *c* without abating even when *c* = 10. The low accuracy for both sibship and parentage inferences when *c* is low is because marker information is rather limited, and is not because the error model is inadequate. With *c* = 1, the dropout rate, 0.75, is so high that only 25% of heterozygotes are observed as such. At this high error rate, 100 SNPs simply do not have sufficient information for pedigree reconstruction. Indeed, 1000 SNPs distributed evenly on a chromosome segment of 5 Morgans yield almost perfect reconstructed pedigrees, with Prb(fs|FS) and Prb(po|PO) being 0.999 and 0.936 when *c* = 1, and 1.0 and 1.0 when *c* = 2.

Figure 3 also shows that parentage inference is more affected by false homozygotes (due to dropouts or sampling errors in NGS) than sibship inference. With 100 SNPs, sibship inference accuracy becomes almost plateaued when *c* reaches 2, but parentage inference accuracy is still increasing rapidly even when *c* reaches 10. Similarly, parentage inference is also more sensitive to false allele rates than sibship inference (Fig. 1).

### Number of SNPs with high false allele rates

**Fig. 4:**
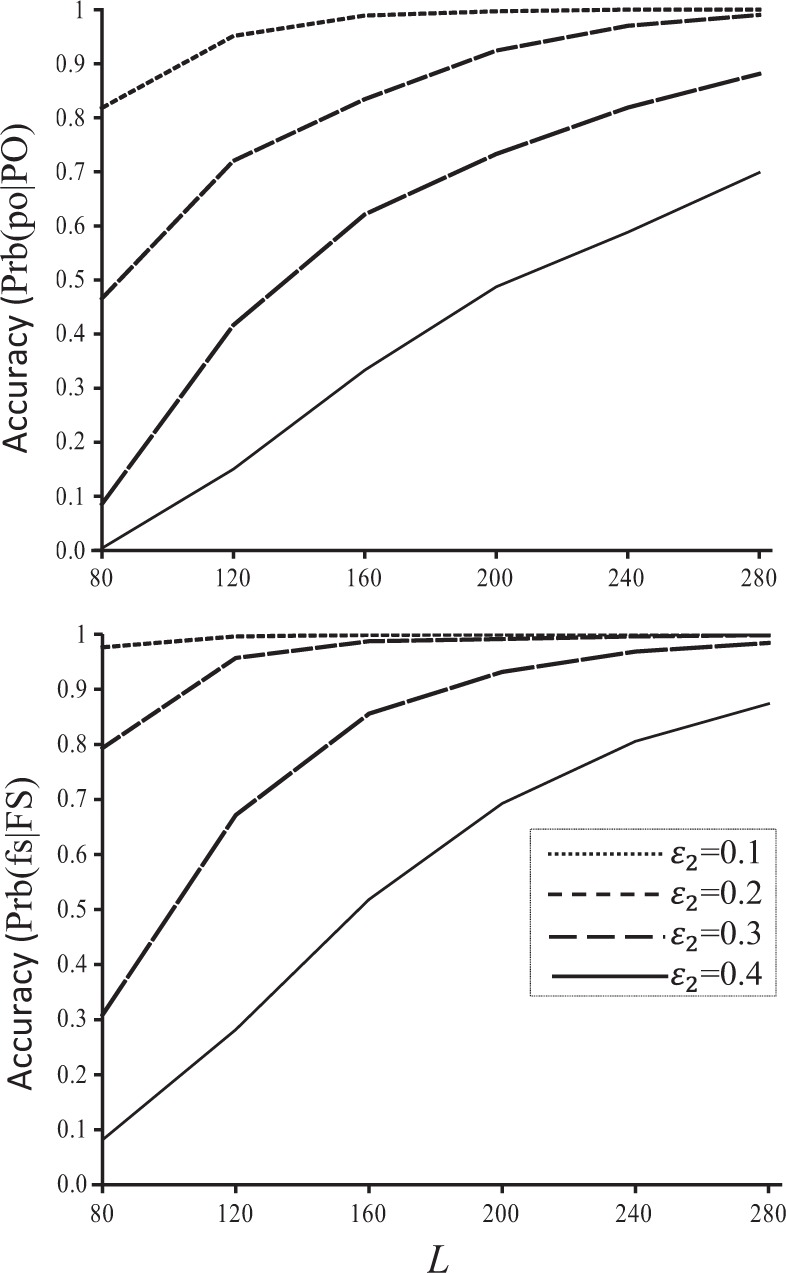
Sibship (Prb(fs|FS), lower panel) and parentage (Prb(po|PO), upper panel) inference accuracy as a function of the number of SNPs (*L*, *x-* axis). A variable number of unlinked SNPs, each with two alleles in a uniform frequency distribution, were used in reconstructing the simulated full-sib pedigree. The dropout error rate was fixed at *ε*_2_ = 0.01 while the false allele rates simulated and used were *ε*_2_ = 0.1, 0.2, 0.3, and 0.4 for each locus. Prb(ns|NS) and Prb(un|UN) are always close to 1 and are thus not plotted for clarity

The new false allele model gives increasingly more accurate inferences of both full sibship and parentage with an increasing number of SNPs of different high false allele rates (Fig. 4). This again proves that markers, even at a high false allele rate of 0.4, are still informative for pedigree reconstruction and more markers lead to more accurate inferences.

### Variation of false allele rates among loci

**Fig. 5:**
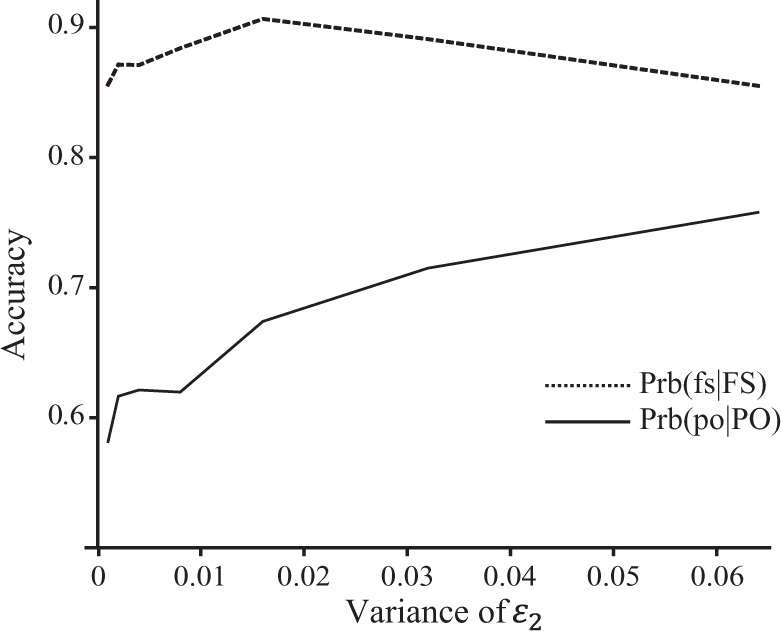
Sibship and parentage inference accuracy (Prb(fs|FS) and Prb(po|PO), *y-*axis) as a function of the variance of *ε*_2_ among loci (*x-*axis). A number of 100 SNPs, each with two alleles in a uniform frequency distribution, were used in reconstructing the simulated full-sib pedigree. The dropout error rate was fixed at *ε*_1_ = 0.01 for each locus, while the simulated and used false allele rate, *ε*_2_, was drawn from a beta distribution with a mean of 0.2 and a variance shown on the *x-*axis. Prb(ns|NS) and Prb(un|UN) are always close to 1 and are thus not plotted for clarity

The inferences of FS and PO relationships are affected differently by the variation in *ε*_2_ among loci (Fig. 5). With an increasing variance of *ε*_2_ in a beta distribution, Prb(fs|FS) is almost constant while Prb(po|PO) increases. At a high variance (say, 0.06), many loci have either high *ε*_2_ values (say, >0.8) or low *ε*_2_ values (say, <0.05). At a low variance (say, 0.002), all loci have *ε*_2_ values closely centred around the mean of $\overline {\varepsilon _2} = 0.2$. It seems that markers of low false allele rates are more important in accurately inferring parentage than sibship.

### Ant data

**Fig. 6:**
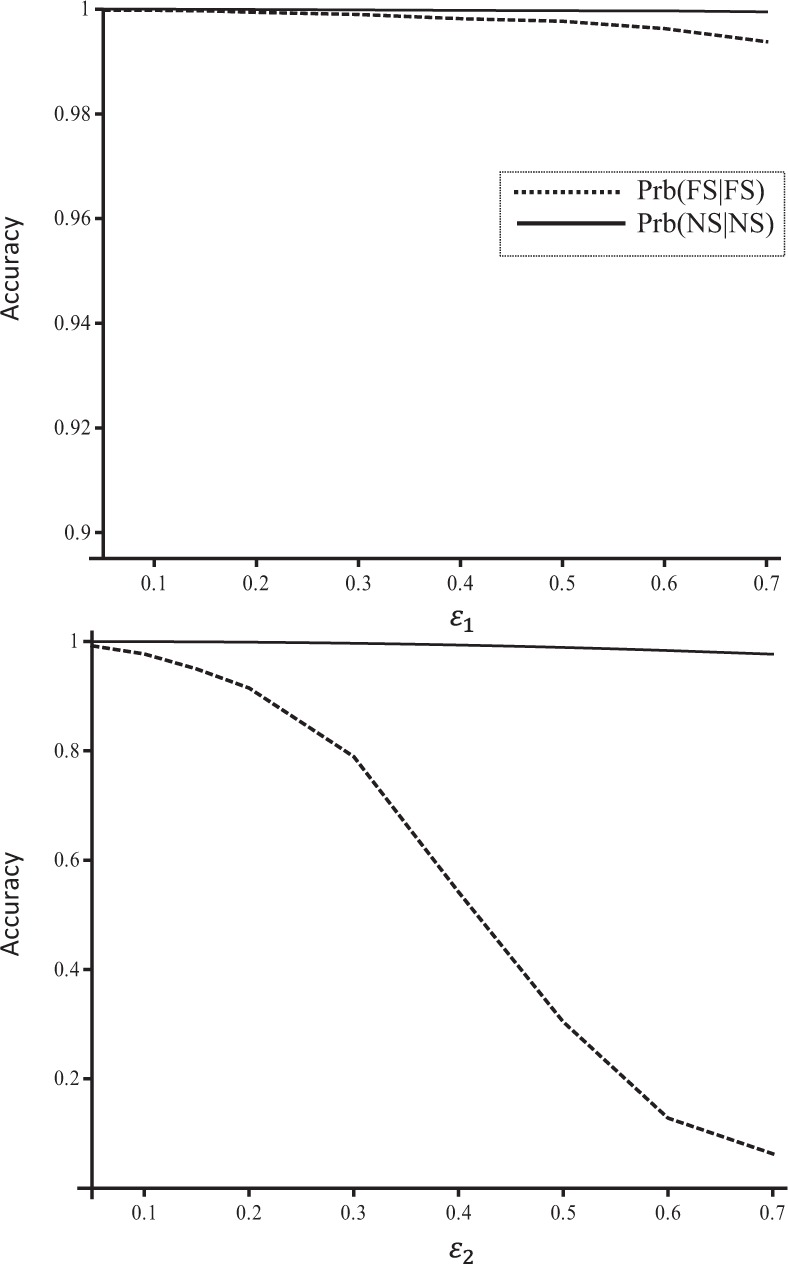
Sibship inference accuracy (Prb(fs|FS) and Prb(ns|NS), *y-*axis) as a function of mistyping rate (*x-* axis) in the ant dataset. Allelic dropouts (upper panel) and false alleles (lower panel) were added to the genotype data according to the error models and error rates (*x-*axis) before the data were analysed for pedigree reconstruction. The results were shown for the modified false allele model, but similar results were obtained from the old model

Allelic dropouts have little effect on the accuracy of reconstructed sibship (Fig. 6). This is true even when dropout rate is extremely high (*ε*_1_ = 0.7). However, sibship inference is affected substantially by false alleles, even when they occur at a moderate rate (say, *ε*_2_ = 0.2).

The results were shown for the modified false allele model, but similar results were obtained from the old model. This is because the two models differ substantially in accuracy only when false allele rate is high and the number of loci is large. This dataset of only six microsatellites with many missing genotypes has simply very little information for pedigree reconstruction when false allele rate is high.

## Discussion

This study shows that how to properly model genotyping errors becomes important in marker-based pedigree reconstruction when such errors occur at a high rate, and when the number of markers is not small. Some error models in the literature (e.g. Sancristobal and Chevalet 1997; Sieberts et al. 2001; Sobel et al. 2002; Wang 2004) work well when error rate is low, but become increasingly inappropriate and inadequate with an increasing error rate. The fundamental problem of these models is that the uncertainty of a phenotype can counterintuitively decrease when error rate approaches its maximal value of 1. The problem becomes more acute with a decreasing polymorphism (i.e. number of alleles or genotypes) at a locus, and with an increasing number of marker loci. It also shows that a modified error model reduces the problem and leads to more accurate sibship and parentage assignments even when error rates are very high.

Integrating proper error models, the likelihood method implemented in Colony (Wang 2004; Wang and Santure 2009) can yield highly accurate sibship and parentage assignments even when markers of the same high error rates are used, as shown in Figs. 1–4. This is especially true for allelic dropouts, and almost perfect relationship inferences could be obtained with either a moderate number of SNPs from very low-coverage sequencing (i.e. high dropout rate, ~0.7) (Fig. 3), or a few microsatellites of high dropout rate (Fig. 6). However, the method is less tolerant to false alleles, especially when few markers are available (Fig. 6). At a high false allele rate (say *ε*_2_ = 0.64), many markers are needed to obtain reasonably good parentage and sibship assignments (Figs. 1, 4). When just a few markers are used, a moderate rate of false alleles can still cause a substantial loss of inference accuracy (Fig. 6). This is because allelic dropouts do not change allele frequencies, but false alleles could homogenize allele frequencies. At a high value of *ε*_2_ at a locus with *k* alleles, the observed allele frequencies calculated from the sampled genotypes (assuming no genotyping errors) would converge to 1/*k*, irrespective of the true underlying allele frequencies. This biased allele frequency estimate calculated and used by Colony would mislead to suboptimal reconstructed pedigrees. Although it could iteratively refine allele frequencies by using the reconstructed pedigrees, genotype data, and genotyping error rate data, this ability is rather limited because it relies on sufficient information to reconstruct the pedigree with few errors. When *ε*_2_ is high and the number of markers is low, the reconstructed pedigree is always far from the truth and thus the refined allele frequencies are also far from the true values. As a result, all inferences (including pedigree, allele frequencies, etc.) are grossly erroneous.

An implication for practical marker-based pedigree analysis is that markers with high error rates should still be used rather than discarded, if the error rates can be estimated. As shown in this study, markers with an allelic dropout rate as high as 0.7 or a false allele rate as high as 0.5 are still informative about genealogy and contribute to the reconstruction of pedigrees. This is great news for situations where high-quality genotype data are out of reach (e.g. microsatellite genotyping from poor noninvasive samples), and where cost and other considerations override data quality (e.g. SNPs from low-coverage NGS). This is also good news for the situation where most loci can be genotyped accurately but a few suffer from a high rate of errors. For example, some microsatellites could have a high null allele frequency or a high dropout rate (due to the use of primers developed for closely related species, for example), even when high-quality DNA extracted from fresh tissue or blood samples is used in PCR. In such situations, genotype data of the problematic loci should still be used in pedigree reconstruction; discarding the data is unnecessary and could cause a substantial loss of precious information.

The new false allele model is implemented in the current version (2.0.6.5) of Colony software downloadable from https://www.zsl.org/science/software/colony.

## References

[CR1] Bonin A, Bellemain E, Bronken Eidesen P, Pompanon F, Brochmann C, Taberlet P (2004). How to track and assess genotyping errors in population genetics studies. Mol Ecol.

[CR2] Douglas JA, Skol AD, Boehnke M (2002). Probability of detection of genotyping errors and mutations as inheritance inconsistencies in nuclear-family data. Am J Human Genet.

[CR3] Ehm MG, Kimmel M, Cottingham RW (1996). Error detection for genetic data, using likelihood methods. Am J Human Genet.

[CR4] Gagneux P, Woodruff DS, Boesch C (1997). Microsatellite scoring errors associated with noninvasive genotyping based on nuclear DNA amplified from shed hair. Mol Ecol.

[CR5] Griffith SC, Owens IPF, Thuman KA (2002). Extra pair paternity in birds: a review of interspecific variation and adaptive function. Mol Ecol.

[CR6] Hadfield JD, Richardson DS, Burke T (2006). Towards unbiased parentage assignment: combining genetic, behavioural and spatial data in a Bayesian framework. Mol Ecol.

[CR7] Hammond RL, Bourke AFG, Bruford MW (2001). Mating frequency and mating system of the polygynous ant, *Leptothorax acervorum*. Mol Ecol.

[CR8] Hauser L, Baird M, Hilborn RAY, Seeb LW, Seeb JE (2011). An empirical comparison of SNPs and microsatellites for parentage and kinship assignment in a wild sockeye salmon (Oncorhynchus nerka) population. Mol Ecol Resour.

[CR9] Jones O, Wang J (2010). COLONY: a program for parentage and sibship inference from multilocus genotype data. Mol Ecol Resour.

[CR10] Marshall TC, Slate J, Kruuk LEB, Pemberton JM (1998). Statistical confidence for likelihood-based paternity inference in natural populations. Mol Ecol.

[CR11] Nielsen R, Paul JS, Albrechtsen A, Song YS (2011). Genotype and SNP calling from next-generation sequencing data. Nat Rev Genet.

[CR12] Ott J (1993). Detecting marker inconsistencies in human gene mapping. Human Hered.

[CR13] Pemberton JM (2008). Wild pedigrees: the way forward. Proc Roy Soc Lond B: Biol Sci.

[CR14] Pompanon F, Bonin A, Bellemain E, Taberlet P (2005). Genotyping errors: causes, consequences and solutions. Nat Rev Genet.

[CR15] Sancristobal M, Chevalet C (1997). Error tolerant parent identification from a finite set of individuals. Genet Res.

[CR16] Sieberts S, Wijsman EM, Thompson EA (2001). Relationship inference from trios of individuals in the presence of typing error. Am J Human Genet.

[CR17] Sobel E, Papp JC, Lange K (2002). Detection and integration of genotyping errors in statistical genetics. Am J Human Genet.

[CR18] Wang J (2004). Sibship reconstruction from genetic data with typing errors. Genetics.

[CR19] Wang J (2006). Informativeness of genetic markers for pairwise relationship and relatedness inference. Theor Popul Biol.

[CR20] Wang J (2018). Estimating genotyping errors from genotype and reconstructed pedigree data. Methods Ecol Evol.

[CR21] Wang J, Santure AW (2009). Parentage and sibship inference from multilocus genotype data under polygamy. Genetics.

